# Screening of dominant strains in red sour soup from Miao nationality and the optimization of inoculating fermentation conditions

**DOI:** 10.1002/fsn3.1992

**Published:** 2020-11-20

**Authors:** Kexin Xiong, Fei Han, Zehan Wang, Ming Du, Yan Chen, Yang Tang, Zhenyu Wang

**Affiliations:** ^1^ National Engineering Research Center of Seafood College of Food Science Dalian Polytechnic University Dalian China

**Keywords:** fermentation conditions, lactic acid bacteria, nitrite, red sour soup, volatile flavor compounds

## Abstract

Red sour soup is a traditional fermented product in southwest China. Currently, the existing production process mainly adopts the method of natural fermentation, with long fermentation cycles and poor stability between batches. Rapid establishment of dominant strains can accelerate the formation of lactic acid, which can inhibit the growth of miscellaneous bacteria. It is also helpful for the inhibition of nitrite accumulation, shortening of fermentation. In this study, the dominant strain H9, with lactic acid‐producing ability, was isolated from the natural fermented red sour soup, and was identified as *Lactobacillus buchneri*, based on the 16s rRNA sequence analysis and biochemical identification. Then, the optimization of fermentation conditions was performed using *L. buchneri* H9 strain as external bacteria. The optimized fermentation conditions were temperature of 22°C, starch dosage of 11.24 g/L, and initial inoculation of 3.5 × 10^8^ cfu/L. The concentration of lactic acid reached 8.029 g/L after 8 days of inoculating fermentation, which exceeded 6.221 g/L for 20 days of natural fermentation. Compared with natural fermentation, the peak of nitrite during inoculating fermentation appeared earlier and the peak height was lower. While the nitrite content in inoculating fermentation decreased to safety threshold more quickly. The volatile flavor compounds analysis showed that 41 types of volatile compounds were detected in the inoculating fermentation product, while 45 in the natural fermentation product. Over 88% compounds were overlapped, which means similar flavor between two fermentation products. These results provide a sufficient scientific basis for the industrialized production of inoculating fermentation of red sour soup.

## Graphical abstract

An acid‐producing dominant strain H9 was screened from the red sour soup, and was identified as *Lactobacillus buchneri*. The inoculating fermentation conditions of red sour soup were optimized using *L. bucheneri* H9 as external starter. The article provide a feasibility for the rapid fermentation of red sour soup using *L. bucheneri* H9 as external starter, under the premise of flavor fidelity.

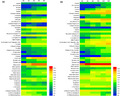

## 1 INTRODUCTION

Red sour soup is a traditional fermented product of the Miao nationality in southwest China. It is usually produced by natural fermentation of a mixture, including tomatoes, red cayenne, glutinous rice flour, white wine, and other ingredients (Li et al., 2016). The soup is rich in a variety of organic acids, minerals and other nutrients, and plays an important role in maintaining nerve and muscle excitability and maintaining the body's acid‐base balance (Byun et al., 2013). At present, it is widely used in the production of sour soup fish, hotpot seasoning and other foods, showing huge market demand.

The fermentation of red sour soup is a process in which the acidity rapidly rises in the early stage and then maintains stable with the production of a variety of volatile flavor substances. Various bacteria groups play an important role in the fermentation process. The formation of flavor substances depends on the synergy between the different bacteria. However, excessive growth of miscellaneous bacteria leads to nitrite accumulation in the early stage of fermentation (Yan et al., 2008). Thus, the most important organic acid is lactic acid, whose accumulation can significantly inhibit the growth of miscellaneous bacteria, and can decrease the concentration of nitrite (Jung et al., 2014). A series of researches on fermented fruits and vegetables showed that the dominant bacteria group was *Lactobacillus*; however, little research has been done on the dominant bacteria in the red sour soup. At present, the production of red sour soup is mostly natural fermentation, whose cycle ranges from 30 days to 1 year (Li et al., 2017). Furthermore, it also has to face the problem of unstable quality between batches, due to the absence of sterilization operation.

Therefore, in this study, the dominant strain with good lactic acid production capacity in natural fermentation was firstly screened and identified at the level of physiology, biochemistry, morphology, and molecular biology. Then, the dominant strain was used as external bacteria for the fermentation of red sour soup, in order to establish an inoculating fermentation system for rapid production of red sour soup. The orthogonal analysis method was used to optimize the fermentation temperature, the starch dosage, and the amount of inoculation to obtain the best inoculating fermentation conditions. The microorganisms, lactic acid contents, pH values, and nitrite contents were determined to reveal changes during the fermentation process and to evaluate the end of inoculating fermentation. Besides, headspace solid‐phase microextraction–gas chromatography‐mass spectrometry was used to determine the concentration of volatile compounds to evaluate the flavor fidelity performance of inoculating fermentation for the production of red sour soup. Through the above research, sufficient scientific basis is provided for the rapid and industrial fermentation for producing the red sour soup.

## 2 MATERIALS AND METHODS

### 2.1 Sample collection

In this study, the naturally fermented red sour soup was collected from a sour soup casserole restaurant on No. 72, Ningbo Road, Kaili, Guizhou Province, southeast China. 57.2% (w/w) of tomatoes and 37.2% (w/w) of red peppers were thoroughly shredded and then mixed with 0.9% (w/w) of glutinous rice flour, 2.6% (w/w) of salt, and 2.1% (w/w) of white wine. The mushy mixture was encapsulated in an aseptic jar and was natural fermented at room temperature (about 17°C) for 20 days. The final production was collected and consequently frozen to −80°C for microorganisms screening and chemical analysis (Monika et al., 2017).

### 2.2 Screening of dominant strains

The samples of red sour soup (1.0 g) were thoroughly mixed with sterile water, forming a 10 ml of suspension. After being left to stand for 10 min at room temperature, the upper suspension was diluted into three different gradients concentration of 10^–4^, 10^–5^, and 10^–6^ (v/v). Hundred microlitre of each suspension was coated on MRS agar medium plate, and incubated at 37°C for 48 hr. After that, strains were selected according to the morphology, color, surface, and edge of the colonies. Each single colony was picked into a new MRS agar medium plate for five generations to obtain the pure culture. These strains were then inoculated in MRS agar medium plate supplemented with 1% of CaCO_3_ (w/v), and the dominant lactic acid‐producing strains were selected by the diameter of transparent circle (mm) and the ratio of transparent circle diameter to colony diameter (Ayodeji et al., 2017).

### 2.3 Molecular biological identification

The colony of screened dominant strain was picked into 2 ml of MRS liquid medium and was cultured at 37°C for 12 hr. The bacterial cell pellet was obtained by centrifugation at 8,000 *g* for 5 min at 4°C (Abid et al., 2018), then its whole genomic DNA was extracted by genomic DNA rapid extraction kit (Biotech Biotechnology Co., Ltd.), and was consequently used as templet for the amplocation of V3–V4 region of 16S rDNA. The PCR reaction was carried out using the reaction program mentioned in Lim's research, with forward primer 27F (5′‐AGAGTTTGATCCTGGCTCAG‐3′) and reverse primer 1492R (5′‐GGTTACCTTGTTACGACTT‐3′) (Lim, 2016; Walter et al., 2000). The gene fragment of V3–V4 region of 16S rDNA was sequenced at Sangon Biotech (Shanghai) Co., Ltd., and the alignment was carried out by blastn project in NCBI GenBank database for molecular biological identification of the strain.

### 2.4 Physiological and biochemical identification

The screened strain was picked up and thoroughly suspended in 2 ml of sterile saline. Then 100 μl of the suspension was added into each well of Lactobacillus Biochemical Identification Kit (Haibo Biotechnology Co., Ltd.), and was cultured at 36 ± 1°C for 12 hr. After that, the color change for each well was observed and compared with specification to determine the utilization of different carbon or nitrogen sources by the strain, including esculin, fiber disaccharides, maltose, mannitol, salicin, sorbitol, sucrose, raffinose, inulin, lactose, and sodium urate (Farahani et al., 2017; Greco et al., 2005; Rizqiati et al., 2015).

### 2.5 Morphological observation

The screened strains were diluted and then coated on MRS agar medium plate, incubating at 37°C for 48 hr. A single colony was picked up from the petri dish and air‐dried on a copper plate for 24 hr. The morphology of the bacteria was observed with a scanning electron microscope (Padmavathi et al., 2018).

### 2.6 Optimization of inoculating fermentation conditions for red sour soup production

The screened strain was inoculated in 100 ml of MRS liquid medium at 37°C, 100 rpm for 24 hr, and the bacterial cells were collected by centrifugation at 1,300 *g* for 10 min. The pellet was washed twice with 15 ml of PBS and was centrifuged at 1,300 *g* for 10 min (Tian et al., 2018). The cells were resuspended in 15 ml of PBS, using as external bacteria for inoculating fermentation. The raw materials in red sour soup inoculating fermentation were prepared including 100 g of thoroughly shredded tomatoes, 230 g of shredded red peppers, 7.5 g of ethanol, and waxy rice starch with different contents, while a mushy mixture including 100 g of shredded tomatoes, 230 g of shredded red peppers, 7.5 g of ethanol, and 6.2 g of waxy rice starch for natural fermentation. The optimization of fermentation temperature, amount of starch dosage, and the amount of inoculum for inoculating fermentation was investigated, with the factor levels showing in Table 1. After 8 days of fermentation, the lactic acid content of each group was used as an evaluation index to obtain the optimal fermentation conditions.

**Table 1:** Factors and levels of orthogonal test

Levels	Factors		
	A (Temperature, °C)	B (Starch dosage, g/L)	C (Inoculum, ×10^9^ cfu/L)
−1	12	1.02	0.35
0	17	6.15	1.75
1	22	11.24	3.15

### 2.7 Chemical analysis

Both inoculating fermented and naturally fermented red sour soup samples were centrifuged at 10,000 *g*, 4°C for 20 min, and the pH of supernatant was tested with a pH meter (Sartorius PB‐10).

For nitrite determination, the supernatants were diluted 10 times (w/v) with ddH_2_O, and then the nitrite content was determined using a colorimetric nitrite assay as described previously. (Belgacem et al., 2010).

For the determination of lactic acid content, the supernatants were diluted 1,000 times (w/v) with ddH_2_O, followed by desalination using a SPE C18 column (Agela Technologies) (Cevasco, et al., 2011). 1.0 ml of desalted sample was injected into high‐performance ion chromatography (HPIC; Thermo Fisher Scientific, DIONEX ICS‐5000 + DP/ DC) using chromatographic conditions as follows: column of Dionex IonPac As23 (4 × 250 mm), guard column of Dionex IonPac AG23 (4 × 50 mm), suppressor Dionex ASRS 300 (4 mm), injection volume 25 μl, and mobile phase containing 4.5 mM of Na_2_CO_3_ and 0.8 mM of NaHCO_3_ (Feng et al., 2015; Milagres et al., 2012) .

### 2.8 Microbial analysis

Brine samples obtained from both traditional fermentation and inoculation fermentation of red sour soup were withdrawn aseptically from the jars and were serially diluted into 10^–6^, 10^–7^, and 10^–8^ (v/v). Plate counts of total microbes and lactic acid bacteria (LAB) were determined. Plate count agar was used for total microbial counts, while MRS agar and MRS with 1% CaCO_3_ (w/v) agar for LAB counts after the plates were incubated at 37°C for 48 hr(Liu et al., 2015).

### 2.9 Determination of volatile compounds

Each fermented red sour soup sample (0.1 g) was added into a headspace bottle for SPME, under the condition of water bath at 50°C for 20 min, extract needle adsorption for 30 min, and 20 μl of cyclohexanone as internal standard (Li et al., 2018; Tang et al., 2018). The volatile flavor compounds were determined using gas chromatography‐mass spectrometry (Agilent Technologies). The conditions for determination were set as described previously (Duffin et al., 2017; Jiang et al., 2017).

### 2.10 Statistical analysis

The experimental data were all three groups in parallel. Statistical analysis was performed on the experimental data using SPSS data processing software. A line chart of the lactic acid content during fermentation was drawn by Origin 8.5 (OriginLab Corporation). The peak area in the volatile flavor compound table is the average of three measurements. The method is mass spectrometry identification combined with RI value comparison (Walter et al., 2000).

## 3 RESULT

### 3.1 Dominant strains of natural fermented red sour soup

After purification of the strains using MRS agar medium plates, the single colonies were coated on MRS agar medium plates containing 1% of CaCO_3_, respectively. A transparent circle will be generated around the colony, which means that the strain could produce acid to degrade its ambient calcium carbonate. The size of the transparent circle is considered to be an important indicator of the acid production ability of the strain (Hwanhlem et al., 2011). The diameters of the transparent circle and strain colonies were shown in Table 2, with the lactic acid production of each strain in MRS liquid medium. It is shown that strain H1–H5 showed high ratio values with good lactic acid yield range from 1.986 ± 0.021 g/L to 2.682 ± 0.012 g/L, while strain H9 had the highest lactic acid yield although its ratio value was only 1.50. Strain H11–H20 showed lower ratio values, which echoed their lower lactic acid yield range from 0.652 ± 0.025 g/L to 1.369 ± 0.041 g/L. It can be seen that the ability of the strain secreting lactic acid is not exactly the same as the size of the transparent circle. It is likely to be affected by the dispersion of lactic acid on the MRS agar, the tolerance of strains to lactic acid accumulation, and the feedback inhibition of lactic acid accumulation on the metabolic pathways of lactic acid production (Hwanhlem et al., 2011). Based on a comprehensive comparison of the size of transparent circle and the lactic acid production, strain H9 was selected for subsequent identification and inoculating fermentation.

**Table 2:** Acid production capacity of the strains

Strain	Colony diameter (d, cm)	Transparent circle diameter (D, cm)	Ratio (D/d)	Lactic acid production (g/L)
H1	0.18 ± 0.01	0.40 ± 0.01	2.22 ± 0.06	2.682 ± 0.012
H2	0.17 ± 0.02	0.40 ± 0.02	2.35 ± 0.07	2.453 ± 0.022
H3	0.20 ± 0.02	0.40 ± 0.01	2.00 ± 0.06	2.091 ± 0.036
H4	0.14 ± 0.01	0.30 ± 0.01	2.14 ± 0.06	1.986 ± 0.021
H5	0.15 ± 0.01	0.35 ± 0.02	2.33 ± 0.07	2.810 ± 0.025
H6	0.29 ± 0.02	0.41 ± 0.02	1.41 ± 0.04	2.121 ± 0.015
H7	0.30 ± 0.02	0.40 ± 0.01	1.33 ± 0.04	0.909 ± 0.016
H8	0.30 ± 0.02	0.33 ± 0.01	1.10 ± 0.04	1.588 ± 0.020
H9	0.30 ± 0.02	0.45 ± 0.02	1.50 ± 0.03	3.268 ± 0.016
H10	0.28 ± 0.02	0.33 ± 0.01	1.18 ± 0.04	2.085 ± 0.036
H11	0.20 ± 0.01	0.20 ± 0.01	1.00 ± 0.03	0.753 ± 0.064
H12	0.21 ± 0.02	0.23 ± 0.01	1.10 ± 0.02	1.369 ± 0.041
H13	0.20 ± 0.01	0.21 ± 0.01	1.05 ± 0.03	1.125 ± 0.036
H14	0.21 ± 0.01	0.23 ± 0.01	1.10 ± 0.03	0.908 ± 0.046
H15	0.20 ± 0.01	0.21 ± 0.01	1.05 ± 0.03	0.875 ± 0.051
H16	0.25 ± 0.02	0.28 ± 0.02	1.12 ± 0.04	0.652 ± 0.025
H17	0.25 ± 0.02	0.26 ± 0.02	1.04 ± 0.02	0.683 ± 0.034
H18	0.24 ± 0.01	0.26 ± 0.01	1.08 ± 0.03	0.798 ± 0.069
H19	0.25 ± 0.01	0.27 ± 0.01	1.08 ± 0.04	0.665 ± 0.042
H20	0.23 ± 0.01	0.25 ± 0.02	1.09 ± 0.03	0.742 ± 0.025

**Figure 1:**
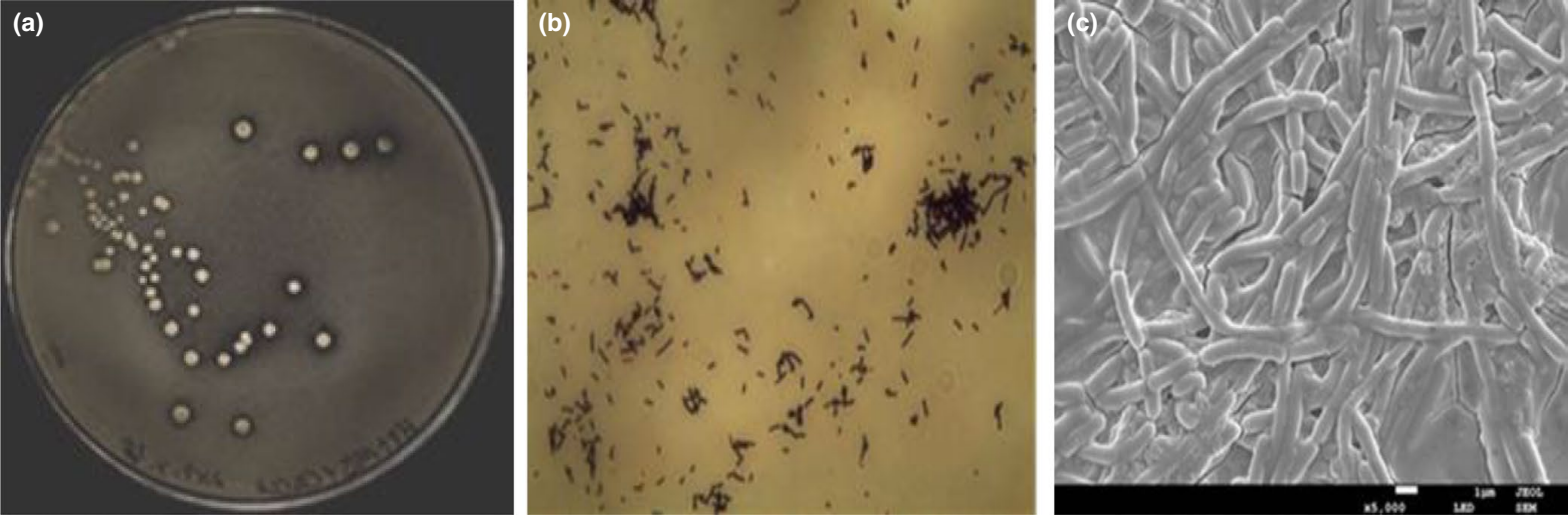
The colonial morphology with transparent circle (a), Gram staining (b), and cellular morphology (c) of dominant strain H9

### 3.2 Identification of dominant strain H9

For molecular biology identification, nucleic acid sequence of 16S rRNA V3–V4 from strain H9 was determined and submitted to the NCBI GenBank database (Accession number: MT410563). The results of nucleotide BLAST (Basic local alignment search tool) alignment showed that 99.12% of 16S rRNA nucleotide sequence identity between strain H9 and *Lactobacillus buchneri* strain Lbu‐07, which strongly suggested that strain H9 might be *L. buchneri*. Further identification of biochemical analysis and morphological observation was subsequently launched.

The colonies of the strain H9 on the MRS agar medium plate were milky white, round, and raised. The edge of them was neat, while the surface was wet and smooth, without wrinkles. Results of Gram staining showed that strain H9 was Gram‐positive. Scanning electron microscope observation showed that the bacterial cells were rod‐shaped, with the size of about 4 μm × 0.7 μm, and tended to aggregate into a chain (Figure 1).

**Figure 2:**
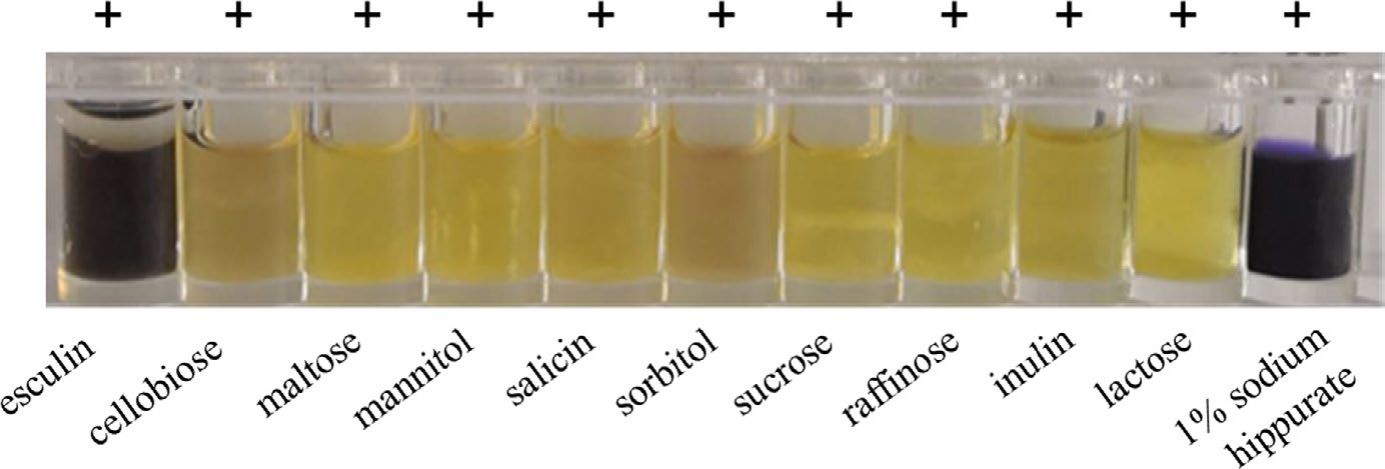
The results of biochemical identification of strain H9

The results of LAB biochemical identification test showed that all the results were positive (Figure 2), which suggested strain H9 to be *L. buchneri*. Combined with the above results, the strain H9 was named *L. buchneri* H9. It was reported that *L. brucei* is a LAB capable of heterogeneous lactic acid fermentation, and it can also perform acetic acid fermentation in an aerobic environment. In addition, *L. buchneri* was widely used for fermented food production or non‐colonized probiotics (Cheon et al., 2020). This provides a very good prospect for the application of *L. brucei* in the fermentation process of red sour soup. Thus, *L. buchneri* strain H9 was applied to subsequent inoculating fermentation experiments.

### 3.3 The optimized inoculating fermentation conditions

Orthogonal experiments were performed to optimize three factors of inoculating fermentation: fermentation temperature, starch dosage, and amount of inoculum. As can be seen from Table 3, all the factors could significantly affect the yield of lactic acid in inoculating fermentation. Relationship of k_A3_>k_A2_>k_A1_ means that as the increasing of fermentation temperature, the lactic acid production of the fermentation increased. It may be due to a rapider growth of LAB under a higher fermentation temperature. On the basis of its own quantitative advantages in the early stage of inoculating fermentation, LAB can inhibit the utilization of carbohydrates by miscellaneous bacteria and convert carbohydrates to produce lactic acid more efficiently. The influence of starch dosage and inoculum amount in the fermentation system on lactic acid production is not the same as the fermentation temperature, which does not show a linear relationship. When the starch dosage reached the B3 level of 11.24 g/L, the results showed the highest average lactic acid production, which may be due to abundant of carbohydrates provide a rich substrate for the production of lactic acid. While, the amount of inoculum at the C1 level (3.5 × 10^8^cfu/L) resulted in the highest average lactic acid production. It is noteworthy that the increase in the amount of initial inoculum did not increase the lactic acid production, which is worthy paying attention to in actual inoculating fermentation. According to the Range (R) value, the most important factor affecting inoculating fermentation was fermentation temperature (A), followed by the amount of starch dosage (B), and the least influential factor was the amount of inoculum (C). According to the analysis of orthogonal test, the optimal fermentation conditions were as follows: fermentation temperature of 22°C (A3), starch dosage of 11.24 g/L (B3), and inoculum amount of 3.5 × 10^8^ cfu/L (C1).

**Table 3:** Results of orthogonal test of fermentation conditions

No.	Experimental factors and levels			*x* (Lactic acid production, g/L)
	A (Temperature, °C)	B (Starch dosage, g/L)	C (Inoculum,×10^9^cfu/L)	
1	12	1.02	0.35	1.375
2	12	6.15	1.75	0.474
3	12	11.24	3.15	2.537
4	17	1.02	1.75	2.694
5	17	6.15	3.15	3.578
6	17	11.24	0.35	7.373
7	22	1.02	3.15	5.358
8	22	6.15	0.35	4.625
9	22	11.24	1.75	5.491
*K*_1_ = Σ*x_i_*_1_ ^a^	4.386	9.427	13.373	
*K*_2_ = Σ*x_i_*_2_ ^a^	13.646	8.677	8.659	
*K*_3_ = Σ*x_i_*_3_ ^a^	15.474	15.401	11.474	
*k*_1_ = $\overset{‐}{x}$ *_i_*_1_ ^a^	1.462	3.142	4.458	
*k*_2_ = $\overset{‐}{x}$ *_i_*_2_ ^a^	4.549	2.892	2.886	
*k*_3_ = $\overset{‐}{x}$ *_i_*_3_ ^a^	5.158	5.134	3.825	
Range (R)	3.696	2.241	1.572	

^a^ *i* = A, B, or C.

**Figure 3:**
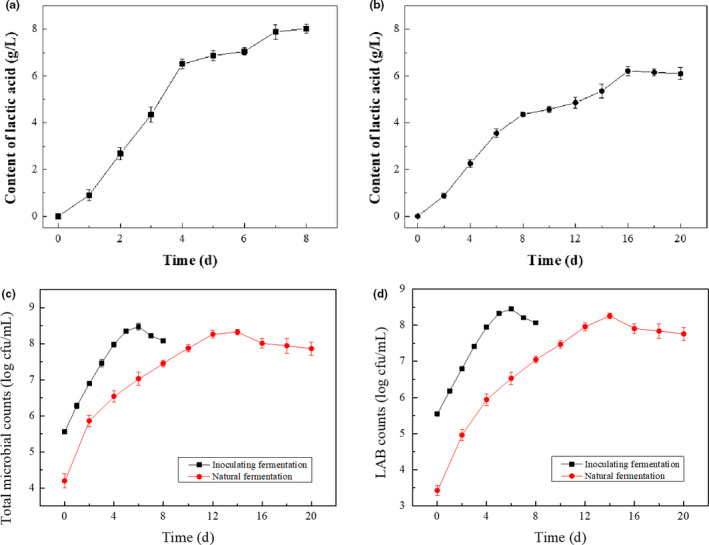
Changes in lactic acid production, total microbial counts, and LAB counts during fermentation. (a) Lactic acid in inoculating fermentation, (b) Lactic acid in natural fermentation, (c) Total microbial counts, and (d) LAB counts

Since the optimal fermentation condition combination was not in the existing orthogonal test combination, validation test for optimal fermentation conditions was carried out, with a natural fermentation as a control. As shown in Figure 3, the lactic acid content increased rapidly within 4 days, and then the growth rate was slightly slower after that. The final concentration of lactic acid reached 8.029 g/L, which was higher than those in all orthogonal test combinations. It was also seen that the lactic acid content of inoculating fermentation increased faster, reaching 6.48 g/L on the 4th day, while the lactic acid content of natural fermentation was only 2.28 g/L on the 4th day. Besides, the final lactic acid concentration of inoculating fermentation after 8 days (8.029 g/L) was much higher than that of natural fermentation after 20 days (6.108 g/L).

The initial total microbial counts were 5.561 log cfu/ml, of which the LAB counts were 5.545 log cgu/ml for the inoculating fermentation system. While, initial total microbial counts were 4.198 log cfu/ml for the natural fermentation system, with LAB counts of 3.421 log cfu/ml. The higher initial number of total microbial counts in inoculating fermentation was due to the introduction of LAB (strain H9) into the culture. In the inoculation fermentation system, the total microbial counts increased rapidly in the first 5 days, and then flattened and reached the maximum of 8.452 log cfu/ml on the 6th day. Thereafter, the total microbial counts began to decrease on the 7th day. In the natural fermentation system, the total microbial counts continued to increase in the first 12 days, and then flattened reaching the maximum of 8.318 log cfu/ml at 14th day. Next to the end of the natural fermentation, the total microbial counts slowly decrease to 7.860 log cfu/ml. It can be seen that in the early stage of fermentation, the rapid growth of LAB and the accumulation of lactic acid content are almost synchronized. The decrease of LAB counts in the later stage will not affect the accumulation of lactic acid content.

It can be seen that inoculating fermentation can accumulate lactic acid faster, which significantly shorten the fermentation period of red sour soup production. Besides, it is widely accepted that faster lactic acid production helps inhibit the growth of miscellaneous bacteria during the early stage of fermentation (Hong et al., 2014), which can also be seen from the difference between the total microbial counts and LAB counts. In the inoculating fermentation system, the initial proportion of LAB reached 96.2%, while it was only 16.7% in the natural fermentation system. As a result, the latter has a higher proportion of non‐LAB and slow accumulation of lactic acid in a longer period of time, which leads to a prolonged product fermentation cycle. Therefore, the method of inoculating fermentation could provide a feasibility for the rapid industrial fermentation of red sour soup.

### 3.4 Changes of pH and nitrite content during inoculating fermentation

**Figure 4:**
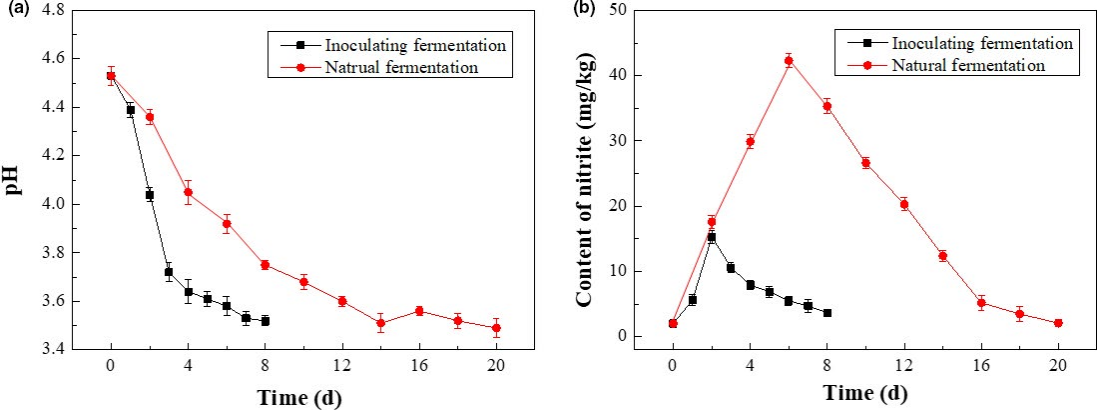
Changes in pH value and nitrite content during fermentations. (a) for pH and (b) for Content of nitrite

pH and nitrite content are important indicators of the change during fermentation process. Using these indicators, the growth rate of LAB and the degree of fermentation of red sour soup can be predicted (Peñas et al., 2010). The change of pH value during inoculating fermentation process was shown in Figure 4a, using pH in natural fermentation as a control. When the fermentation system was initially prepared (0 day), the pH value was 4.53. The pH in inoculating fermentation decreased faster than that in natural fermentation, reaching pH 4.04 within 2 days and pH 3.64 after 4‐day fermentation. It could be interpreted as the rapid growth of LAB within 2 days of inoculating fermentation, resulting in the lactic acid accumulation and pH reduction. After that, the pH remained stable in the range from 3.42 to 3.64 until the end of inoculating fermentation at 8 days. The change of pH value during inoculating fermentation was the same as that of Chinese traditional fermented vegetable *Paocai* (Liu et al., 2015).

Nitrite, harmful to human body, is particularly concerned about the changes in the content of fermented vegetables, the content of which is also an important indicator to evaluate the end of fermentation. The change of nitrite content was shown in Figure 4b. The peak of nitrite concentration in inoculating fermentation (15.3 mg/L on day 2) was significantly lower than that of natural fermentation (42.3 mg/L on day 6). In addition, the peak of nitrite concentration appeared earlier in the inoculating fermentation process. It was reported that the formation of nitrite is closely related to the growth of miscellaneous bacteria in the early stage of fermentation, whose nitrate reductase reduces nitrate into nitrite, resulting in a sharp increase in nitrite content. However, with the large number of LAB growing and taking advantage, the nitrite reductase produced by LAB can quickly degrade nitrite (Yan et al., 2008). In addition, a lower pH (≤4.0) can not only effectively inhibit the growth of miscellaneous bacteria, thereby reducing the content of nitrite, but also achieve the degradation of nitrite through acid degradation reaction (Yang et al., 2014).

According to China national standard GB2762‐2017, the nitrite concentration in fermented vegetables should be less than 20 mg/kg. Thus, combined with changes in lactic acid content, pH value, nitrite content, and the concentration process during red sour soup product processing, it can be concluded that the inoculating fermentation process can be terminated on the 8th day, while the natural fermented process needs to last at least 20 days. The above results demonstrated that inoculating fermentation occurred faster in the presence of strain H9. Adopting the method of inoculating fermentation using strain H9 could therefore result in the production of a food with more commercial appeal.

### 3.5 The volatile compounds

**Figure 5:**
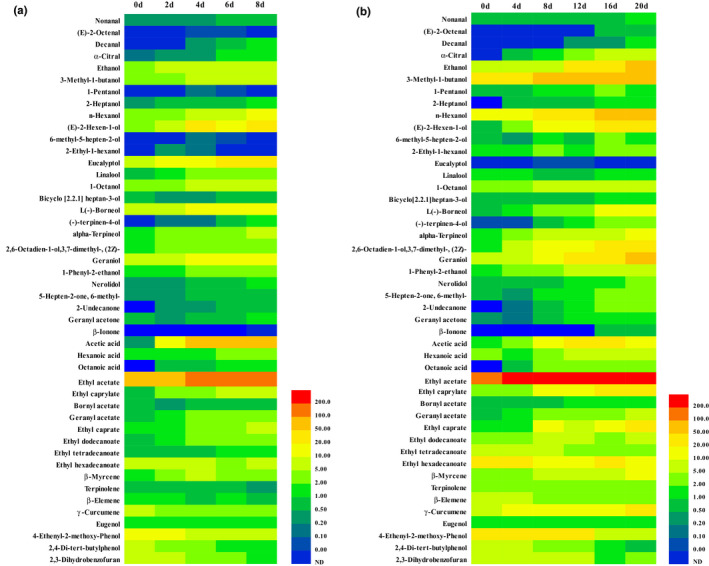
Heat map of volatile flavor compounds during fermentation process. (a) Inoculating fermentation, (b) Natural fermentation

The change in volatile flavor compounds during inoculating fermentation and natural fermentation is shown in Figure 5. The content of most volatile compounds increased gradually along with the fermentation process, while a small number of compounds decreased gradually with the fermentation process, such as β‐elemene, 4‐ethenyl‐2‐methoxy‐phenol, 2,4‐di‐tert‐butylphenol, and 2,3‐dihydrobenzofuran. The contents of some alcohols and esters in the final products were abundant, which also changed significantly during the fermentation process, such as 3‐methyl‐1‐butanol, n‐hexanol, geraniol, and ethyl acetate. Most of the volatile compounds in the natural fermented res sour soup can be also found in the inoculating fermentation. As further shown in Table 4, a total of 41 volatile flavor compounds were detected in inoculating fermented production, including three aldehydes, 16 alcohols, three ketones, three acids, eight esters, four alkenes, and four other kinds of volatile compounds, while a total of 45 volatile flavor compounds were detected in the natural fermented production, including four aldehydes, 18 alcohols, four ketones, three acids, eight esters, four alkene, and four other kinds of volatile compounds. A total of 40 kinds of volatile compounds were overlapped in two different fermentation products, which indicates that the flavor of two products by different fermentation methods may be close. It can also be seen that the contents of volatile flavor compounds in the inoculating fermented red sour soup are generally lower than that in the naturally fermented soup. Thus, the flavor of final inoculating fermented product may not be as strong as the naturally fermented product. These results provide a feasibility for the rapid fermentation of red sour soup under the premise of flavor fidelity.

**Table 4:** Comparison of volatile compounds

Compounds	KI	Identification methods	Inoculating fermentation 8 days	Natural fermentation 20 days
Aldehyde				
Nonanal	1,104	MS RI	0.78	1.45
(E)‐2‐Octenal	1,156	MS RI	–	0.80
Decanal	1,206	MS RI	1.62	1.95
α‐Citral	1,272	MS RI	1.98	6.44
Alcohol				
Ethanol	427	MS RI	7.35	72.82
3‐Methyl‐1‐butanol	786	MS RI	8.19	64.45
1‐Pentanol	831	MSRI	–	1.63
(E)‐2‐Hexen‐1‐ol	862	MS RI	20.58	21.23
2‐Heptanol	901	MS RI	0.56	1.22
n‐Hexanol	934	MSRI	14.25	50.11
6‐methyl‐5‐hepten‐2‐ol	1,016	MSRI	–	1.89
2‐Ethyl‐1‐hexanol	1,051	MSRI	–	3.32
Eucalyptol	1,029	MS RI	23.30	–
Linalool	1,100	MS RI	2.08	1.39
1‐Octanol	1,112	MSRI	5.21	6.98
bicyclo[2.2.1]heptan‐3‐ol	1,148	MS RI	0.68	1.71
L(‐)‐Borneol	1,165	MS RI	10.72	14.22
(‐)‐Terpinen‐4‐ol	1,177	MS RI	1.35	3.88
alpha‐Terpineol	1,190	MS RI	6.90	12.23
2,6‐Octadien‐1‐ol,3,7‐dimethyl‐, (2Z)‐	1,229	MS RI	4.49	37.23
Geraniol	1,255	MS RI	12.77	51.67
1‐Phenyl‐2‐ethanol	1,369	MS RI	2.90	5.67
Nerolidol	1,564	MS RI	1.41	3.13
Ketones				
5‐Hepten‐2‐one, 6‐methyl‐	1,144	MS RI	0.80	1.95
2‐Undecanone	1,294	MS RI	0.59	3.65
Geranyl acetone	1,452	MS RI	1.25	1.86
β‐Ionone	1,672	MS RI	‐	0.58
Acid				
Acetic acid	610	MS RI	97.67	16.24
Hexanoic acid	1,596	MS RI	2.89	6.37
Octanoic acid	1,807	MS RI	1.37	3.74
Ester				
Ethyl acetate	668	MS RI	136.94	299.71
Ethyl caprylate	1,198	MS RI	8.55	23.49
Bornyl acetate	1,286	MS RI	0.52	1.95
Geranyl acetate	1,383	MS RI	4.64	5.74
Ethyl caprate	1,416	MS RI	5.23	22.61
Ethyl dodecanoate	1,612	MS RI	4.97	9.72
Ethyl tetradecanoate	1,816	MS RI	1.41	3.71
Ethyl hexadecanoate	2,022	MS RI	6.21	15.38
Alkene				
β‐Myrcene	992	MS RI	4.10	13.12
Terpinolene	1,088	MS RI	0.40	2.36
β‐Elemene	1,393	MS RI	0.54	2.81
γ‐Curcumene	1,480	MS RI	2.46	25.59
Others				
Eugenol	1,919	MS RI	1.05	1.08
4‐Ethenyl‐2‐methoxy‐Phenol	1,948	MS RI	6.77	8.46
2,4‐Di‐tert‐butylphenol	2,070	MS RI	0.91	0.60
2,3‐Dihydrobenzofuran	2,151	MS RI	1.22	2.28

## 4 CONCLUSION

In this study, the acid‐producing dominant strain H9 was screened from the red sour soup and was identified as *L. buchneri* H9. The inoculating fermentation conditions of red sour soup were optimized using *L. bucheneri* H9 as external bacteria, and the optimal fermentation conditions were temperature of 22°C, starch dosage of 11.24 g/L, and inoculum of 3.5 × 10^8^ cfu/L. Through the detection of lactic acid, pH, and nitrite in two products by different fermentation methods, inoculating fermentation can significantly shorten the production cycle of red sour soup. Analysis of volatile flavor compounds showed that inoculating fermentation could achieve a similar but slightly weaker flavor to natural fermentation. These results provide a feasibility for the rapid fermentation of red sour soup using *L. bucheneri* H9 as external bacteria, under the premise of flavor fidelity.

## CONFLICT OF INTEREST

The author declares no conflict of interest.
